# Estimated Mask Use and Temporal Relationship to COVID-19 Epidemiology of Black Lives Matter Protests in 12 Cities

**DOI:** 10.1007/s40615-022-01308-4

**Published:** 2022-05-11

**Authors:** Ashley Quigley, Phi Yen Nguyen, Haley Stone, David J. Heslop, Abrar Ahmad Chughtai, C. Raina MacIntyre

**Affiliations:** 1 Biosecurity Research Program, The Kirby Institute, UNSW, Wallace Wurth Building, UNSW, High St, Kensington Campus, Sydney, NSW 2052 Australia; 2 School of Population Health, UNSW, Level 3, Samuels Building, UNSW, Sydney, NSW 2052 Australia

**Keywords:** Mass protests, COVID-19, Transmission, Mask use

## Abstract

There is an increased risk of SARS-CoV-2 transmission during mass gatherings and a risk of asymptomatic infection. We aimed to estimate the use of masks during Black Lives Matter (BLM) protests and whether these protests increased the risk of COVID-19. Two reviewers screened 496 protest images for mask use, with high inter-rater reliability. Protest intensity, use of tear gas, government control measures, and testing rates were estimated in 12 cities. A correlation analysis was conducted to assess the potential effect of mask use and other measures, adjusting for testing rates, on COVID-19 epidemiology 4 weeks (two incubation periods) post-protests. Mask use ranged from 69 to 96% across protests. There was no increase in the incidence of COVID-19 post-protest in 11 cities. After adjusting for testing rates, only Miami, which involved use of tear gas and had high protest intensity, showed a clear increase in COVID-19 after one incubation period post-protest. No significant correlation was found between incidence and protest factors. Our study showed that protests in most cities studied did not increase COVID-19 incidence in 2020, and a high level of mask use was seen. The absence of an epidemic surge within two incubation periods of a protest is indicative that the protests did not have a major influence on epidemic activity, except in Miami. With the globally circulating highly transmissible Alpha, Delta, and Omicron variants, layered interventions such as mandated mask use, physical distancing, testing, and vaccination should be applied for mass gatherings in the future.

**Supplementary Information:**

The online version contains supplementary material available at 10.1007/s40615-022-01308-4.

**What is Already Known on This Subject?**

There is an increased risk of SARS-CoV-2 transmission during mass gatherings and a risk of asymptomatic infection. Shouting and subsequent aerosol generation among people in close proximity, use of tear gas, and inability to trace all contacts at mass gatherings are a further risk. This study is necessary to estimate the use of masks during protests and analyze the temporal relationship of protests to COVID-19 epidemiology.

**What This Study Adds?**

The absence of an epidemic surge within two incubation periods of the protests is indicative that the protests did not have a major influence on epidemic activity, except in Miami. Outdoor mass gatherings with high levels of mask use may not pose as high a risk for COVID-19 as indoor gatherings. The risk of Omicron transmission outdoors, however, may be higher.

## Introduction

Late May and early June 2020 saw thousands of people around the world gathering for Black Lives Matter (BLM) mass protests. In light of the COVID-19 pandemic, in which 488 million people have been infected with over 6.14 million deaths [1], there was some concern about the potential detrimental effect of these mass protests on the control of COVID-19, not only from close contact and the inability to social distance, but to the unplanned, increased movement of protestors to and from various cities and states by public transport [2, 3]. Growing evidence supports airborne transmission of severe acute respiratory syndrome coronavirus 2 (SARS-CoV-2), the virus which causes COVID-19 [4–9]. This coupled with the primary transmission route through inhalation of respiratory droplets and close contact, as well as substantial asymptomatic or pre-symptomatic transmission, together with the emergence of the highly infectious Alpha, Delta, and Omicron variants, makes epidemic control challenging [10–14]. Several factors influence SARS-CoV-2 transmission in mass gatherings [15, 16]: the geographic scope of the gathering; event-specific behaviors such as shouting and singing; density and gathering size; and, most importantly, the environment in which the gathering occurs, i.e., indoors or outdoors [17]. Due to the increased risk of SARS-CoV-2 transmission during mass gatherings and the risk of asymptomatic infection [17–19], protestors were urged to follow the mandate released by the Centers for Disease Control and Prevention (CDC), to wear cloth masks in public and physically distance where possible [20]. A mask may prevent outward particle emissions from an infected person, referred to as source control, but masks also protect well people from infection [21]. Effective mask types may not be limited to surgical masks or respirators. Consistent use of a face mask (cloth, surgical) or N95 respirator in indoor public settings has been associated with lower odds of a positive SARS-CoV-2 test result [22]. Masks have also been found to be effective by use of healthy persons in closed community settings to provide protection against respiratory infections [21].

Shouting and subsequent aerosol generation among people in close proximity, and inability to trace all contacts at mass gatherings, are a further risk. Droplet and aerosol generation when singing or shouting combined with sustained viral viability outside the body is a reason why mass masking of protestors at mass gatherings has been recommended [7, 23]. In addition, use of tear gas at protests may result in enhanced aerosol generation and increased susceptibility of the ocular and nasopharyngeal mucosa [24, 25]. Negative sentiment to government instructions about masks is also an issue [26, 27].

This study aimed to estimate the use of masks during mass protests in the COVID-19 pandemic and the temporal relationship and risk factors of protests to COVID-19 epidemiology using photo-epidemiology.

## Methods

### Data Collection

To estimate global mask use in mass gatherings which occurred during the COVID-19 pandemic, several cities where Black Lives Matter protests took place were selected for analysis (Table 1) according to the following inclusion criteria:

**Table 1:** Global and national cities selected for analysis in this study based on the inclusion criteria detailed in Addendum 1. Cities are grouped by their level of protest intensity.

	High intensity	Medium intensity	Low intensity
USA	Miami (Florida)	New York (New York State)	Columbia (South Carolina)
			Dallas (Texas)
UK	London (Greater London)	Dublin (Leinster, Ireland)	–––-
Europe	Berlin (Berlin)	–––-	Lisbon (Estremadura, Portugal)
	Paris (Ile-de-France)		
Australia	–––-	Sydney (New South Wales)	Melbourne (Victoria)
Canada	Toronto (Ontario)	–––-	–––-

We selected cities from countries where:

1. Mask wearing is not a cultural norm
2. With a population of greater than 80,000 people (< small urban areas [28])
3. Which had Black Lives Matters protests between May 25 and June 7, 2020
4. Government restrictions were eased at least 2 weeks before the protest dates
5. Each protest had greater than 500 people in attendance

Cities meeting these criteria and selected for analysis were from Australia, Canada, the United States of America (USA), the UK, and Europe (Table 4, Addendum 1).

Cities were ranked according to protest intensity; cities where only 1 protest occurred during a week were ranked as low intensity, cities where 2–3 protests occurred in 1 week were ranked as medium intensity, and cities where more than 3 protests occurred over a 2-week period was ranked as high intensity. The date the first protests took place, the relevant government stringency index (GSI) score for COVID-19 restrictions at the time of the protests as determined by the Blavatnik School of Government, Oxford University [29], was recorded for each city. The season (summer, winter) and use of tear gas in the protests (based on media reports, Addendum 2) were also recorded.

To estimate mask use amongst protestors in the globally termed “Black Lives Matter Protests,” photo-epidemiology was used [30–32]. A Google search for protest images was conducted between July 20, 2020, and August 20, 2020, using the following keywords: “{London} AND {“george floyd” OR “protests” OR “black lives matter” OR “blm”} after:2020–05-27”; “{Dublin} AND {Leinster or IE-L AND {“george floyd” OR “protests” OR “black lives matter” OR “blm”} after:2020–05-29”; “{Melbourne} AND {Victoria OR VIC} {“george floyd” OR “protests” OR “black lives matter” OR “blm”} after:2020–06-05”; “{Sydney} AND {New South Wales OR NSW} {“george floyd” OR “protests” OR “black lives matter” OR “blm”} after:2020–06-01”; “{Columbia} AND {South Carolina or SC} AND {“george floyd” OR “protests” OR “black lives matter” OR “blm”} after:2020–05-29”; “{Dallas} AND {Texas OR TX} AND {“george floyd” OR “protests” OR “black lives matter” OR “blm”} after:2020–05-28”; “{New York OR NYC} AND {“george floyd” OR “protests” OR “black lives matter” OR “blm”} after:2020–05-27”; “{Miami} And {Florida OR FL} AND {“george floyd” OR “protests” OR “black lives matter” OR “blm”} after:2020–05-29”; “{Toronto} AND {Toronto or TOR} AND {“george floyd” OR “protests” OR “black lives matter” OR “blm”} after:2020–05-29”; “{Paris} AND {il-de-France or IDF} AND {“george floyd” OR “protests” OR “black lives matter” OR “blm”} after:2020–05-29”; “{Berlin} AND {Berlin or BE} AND {“george floyd” OR “protests” OR “black lives matter” OR “blm”} after:2020–05-29”; and “{Lisbon} AND {Estremadura or Portugal} AND {“George floyd” OR “protests” OR “black lives matter” OR “blm”} after:2020–05-29”.

Where applicable, Google Translate was used to source articles in the country language. The first 3 pages of each Google search were reviewed. Images were included for analysis if they fulfilled the following conditions: (1) a cross-sectional image with a minimum of 50 persons, among which at least 20 were facing forward; (2) images were not staged, e.g., a ceremonial photo-op; (3) and images had to have a title that contained the location of the protest or were associated with a media article about the protest that specified the city’s name. A minimum of 20 images per protest were selected for analysis and are provided in the Supplementary Material.

### Data Analysis

For the purpose of crowd counting, manual counting was performed using the part-based detection method, where specific body parts such as the head and shoulder were used to estimate the people counts in a designated area [33, 34]. Protesters were counted in each image where at least 80% of the protester’s head appeared in the image in such a way that was visually reasonable to detect face covering. Protestors were defined as those actively demonstrating, whilst persons observing (i.e., any personnel in uniforms indicative of the police, army, or national guards) were excluded from counting as mask use was mandated for official personnel. Persons were counted as mask-wearing if they were wearing a respirator, surgical mask, cloth mask, or bandana, and only if their nostrils and mouths were covered. As such, persons were excluded from the count if they were wearing these forms of face coverings around their necks or below the nostrils. The percentage of mask users was defined as the number of protesters wearing masks, divided by the total number of protesters counted, expressed as a percentage. Each image was assigned an identification (ID) number and randomly assigned 2 researchers for manual review.

Inter-rater reliability index was assessed using intraclass correlation coefficient (ICC), calculated using a two-way random-effects model. Recounting or any revision to counting procedures was performed until an ICC of at least 0.80 was achieved [35–37]. The mean reported percentage of mask users from both reviewers was used for the correlation analysis.

The ICC shows a high degree of inter-reviewer reliability for results presented in Table 2. Based on 496 photo counts, the ICC for average measures was 0.927 (95% CI 0.907–0.943, *p* < 0.001).

**Table 2:** Intraclass correlation coefficient of 496 images counted for this analysis.

	ICC	95% Confidence Interval		F test with true value 0			Statistical significance
		Lower bound	Upper bound	Value	df1	df2	
Single measures	0.865	0.829	0.893	13.72	247	247	*p* < 0.001
Average measures	0.927	0.907	0.943				

An incidence curve was plotted to describe the distribution of COVID-19 cases for each county/state, to be representative of the selected city and surrounding areas to account for protestors who travelled into the area of the protest. Localized COVID-19 data for each county/state was sourced from the respective county/state health departments for each city (Addendum 2). The number of COVID-19 cases on the day of the protest was used as the baseline rate. COVID-19 incidence at 2 weeks and 4 weeks post-protests (corresponding to one and two incubation periods after the protest) was recorded. Incidence rates were calculated by dividing new COVID-19 cases by the total uninfected population (adjusted for protest size with respect to each city’s overall population to account for city density) multiplied by 100 population for each time interval and were graphed to show distribution. Given that case notification is affected by testing rates, weekly case data was standardized using testing data. For each county/state, the localized case per test from April 6 to July 26, 2020 (weeks 15–30) was calculated. Testing data specific to each city was unavailable. Weeks 22–23 correspond to baseline, i.e., the initial protest date, whilst weeks 24–25 and weeks 26–27 correspond to 1 and 2 viral incubation periods. Weekly COVID-19 testing data were collected from individual county/state health departments (Addendum 2).

Analysis and reporting were based on the Strengthening the Reporting of Observational studies in Epidemiology (STROBE) guidelines for epidemiological studies [38]. The data generated from this study was cleaned prior to analysis and presented using descriptive statistics after analysis with Stata IC version 16.1. The median of the image reviewer counts was used in the statistical analysis. Mask users per 100 protestors, the GSI score, and COVID-19 testing per 1000 persons 4 weeks post-protest were tested against incidence with the use of Pearson’s correlation coefficient analysis [39] (Table 6, Addendum 1). In addition, a linear regression on reported case numbers, compared to testing rates post 4 weeks of the protest, GSI, and mask use, was performed (Fig. 2, Addendum 1).

## Results

Descriptive characteristics of the protests in each city are shown in Table 3. The date the first protests took place, the relevant GSI score [29] for COVID-19 regulations (where 100 is the most stringent) at the date of the protest, and whether tear gas was used are shown. Tear gas was used in 6 (50%) of the cities analyzed. At the date of the first protest, Lisbon, London, and New York had the highest GSI score (> 70), whilst Toronto, Berlin, and Dublin had the lowest GSI score (< 45).

**Table 3:** Descriptive characteristics of protests selected national and global cities where mass protests occurred.

City	State	Country	Easing of government restrictions	Date of first protest	Government stringency index (GSI)	First use of tear gas	Protest intensity	% mask use	Season
Lisbon	Estremadura	Portugal	May 3^rd^	June 6	71.3	––-	Low	96.5	Summer
Melbourne	Victoria	Australia	May 4^th^	June 6	52.78	––-	Low	92.2	Winter
New York	New York	USA	May 15^th^	May 28	74.04	May 28	Medium	91.9	Summer
Toronto	Ontario	Canada	May 19th	May 30	43.52	––-	High	89.7	Summer
Miami	Florida	USA	May 4^th^	May 29	66.67	June 1	High	87.1	Summer
Columbia	South Carolina	USA	May 4^th^	May 29	51.85	May 31	Low	84.1	Summer
Dallas	Texas	USA	April 30^th^	May 28	65.28	May 30	Low	82.1	Summer
Berlin	Berlin	Germany	May 9^th^	May 30	42.13	––-	High	82.1	Summer
London	Greater London	England	May 15^th^	May 28	71.3	June 13	High	79.4	Summer
Paris	Ile-de-France	France	May 11^th^	May 30	59.72	June 2	High	78.3	Summer
Sydney	New South Wales	Australia	May 14^th^	June 2	52.78	––-	Medium	78.3	Winter
Dublin	Leinster	Ireland	May 18^th^	June 1	48.15	––-	Medium	69.1	Summer

The prevalence of mask use per 100 protestors during the global and national protests at baseline is listed in Table 3. Dublin protestors had the lowest frequency of mask users at 69.1%, whilst Sydney (New South Wales), London (Greater London), and Paris (Ile-de-France) had mask use of 78.3%, 79.4%, and 78.3%, respectively. There was over 80% mask use in protesters in each of the remaining cities analyzed, with the highest percentage of mask users (> 90%) seen in protestors in Lisbon (Estramedura), Melbourne (Victoria), and New York (New York).

**Fig. 1:**
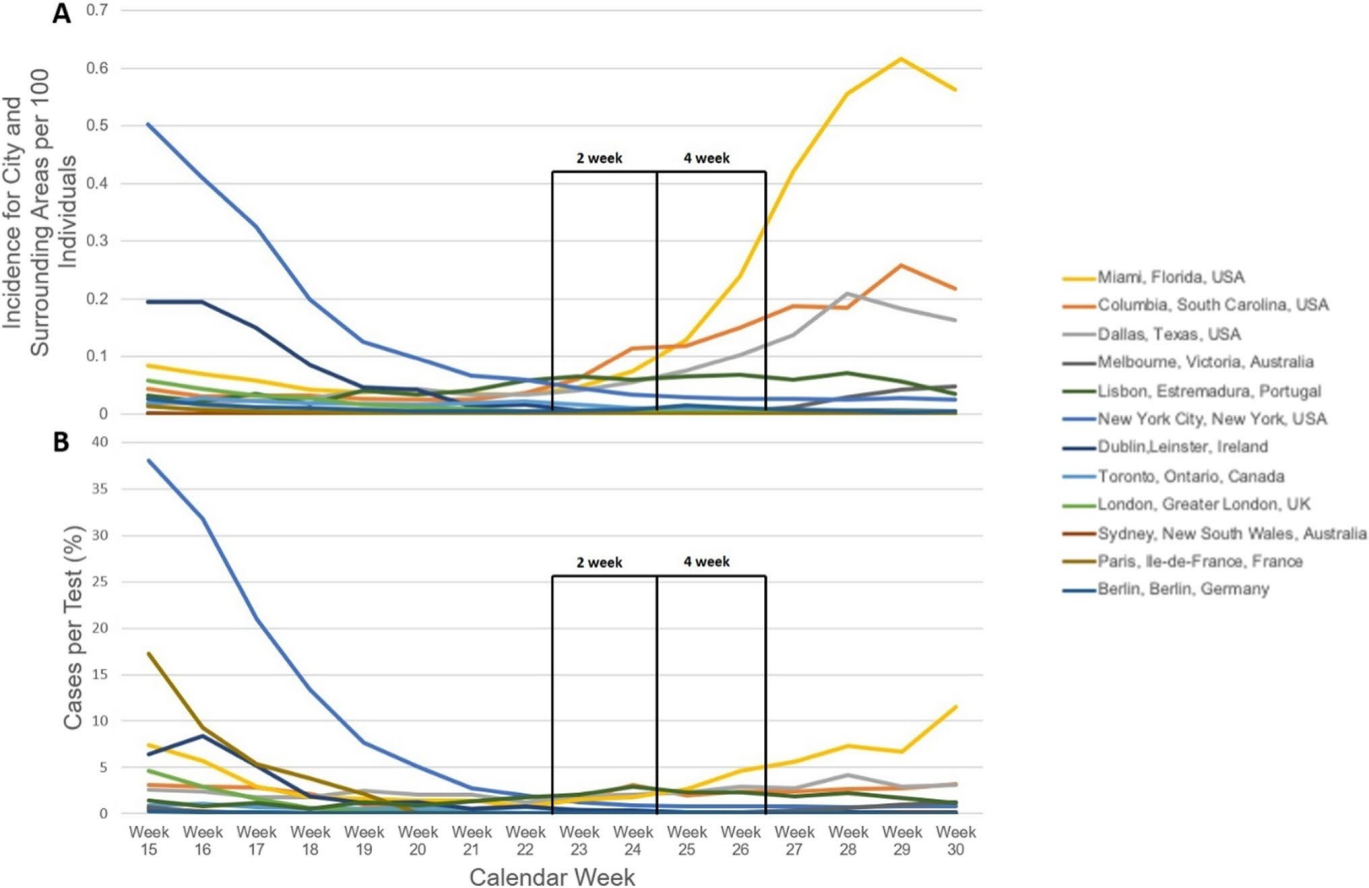
**A** The incidence of COVID-19 in the 12 cities and surrounding areas per 100 individuals. The 2-week and 4-week incubation periods for COVID-19 are indicated. **B** The distribution of COVID-19 cases per test in the 12 cities analyzed expressed as a percentage. The 2-week and 4-week incubation periods for COVID-19 are indicated. Protests occurred in late May 2020 in the USA (weeks 22 and 23), and June 6 for the other cities analyzed (week 23)

**Fig. 2:**
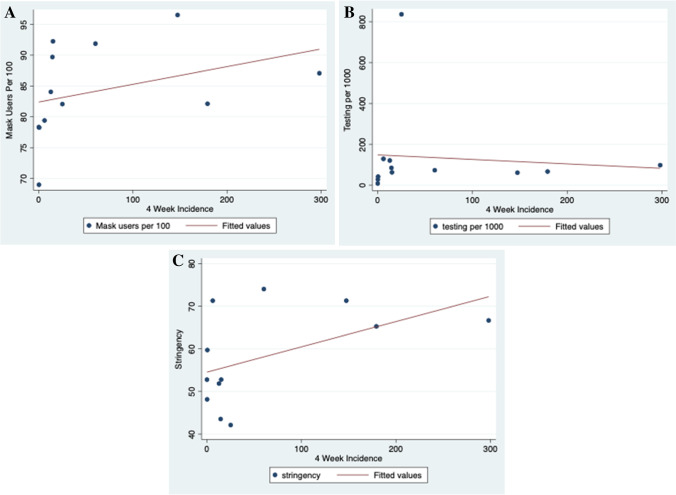
**A** Linear regression between incidence and mask users per 100 (r = 0.1269; 95% CI − 0.0244 to 0.0817; p < 0.256). Each point represents one country. **B** Linear regression between incidence and testing per 1000 persons at 4 weeks post-protest (r = 0.0086, 95% CI − 0.3378 to 0.2588; p < 0.752). Each point represents one country. **C** Linear regression between incidence and GSI (r = 0.2518; 95% CI − 0.0127 to 0.1314; p < 0.096). Each point represents one city

Figure1A and B show the incidence curves of COVID-19 cases reported from April 5 (week 15) to July 26, 2020 (week 30), for the 12 cities analyzed, with the 2- and 4-week incubation periods for COVID-19 post-protest date indicated. Figure 1A shows an increase in COVID-19 incidence between baseline and 2- and 4-week incubation periods after the first protest date (weeks 22–23) in Miami (Florida), Columbia (South Carolina), and Dallas (Texas). A steady progression of COVID-19 incidence was also seen in Lisbon (Estramedura). An increase in COVID-19 incidence in Melbourne (Victoria) was seen after 3 incubation periods of the virus. There was no increase in COVID-19 incidence seen in Sydney (New South Wales), London (Greater London), Toronto (Ontario), Berlin (Berlin), Dublin (Leinster), and Paris (Ile-de-France). Tear gas was used for crowd control in 6 of the 12 protests analyzed, namely, New York (New York), Miami (Florida), Columbia (South Carolina), Dallas (Texas), London (Greater London), and Paris (Ile-de-France).

To account for the impact testing has on identifying COVID-19 cases per test was calculated and is shown in Fig. 1B. Miami (Florida), Columbia (South Carolina), Dallas (Texas), Berlin (Berlin), and Melbourne (Victoria) all had increase in cases per test following the week of protest; however, the standardized data indicated less of an increase in cases/test for Melbourne (Victoria) and Columbia (South Carolina). Miami (Florida) and Dallas (Texas) still exhibited an upward trend in COVID-19 cases/test following both 2 and 4 virus incubation periods. The trend for cases per test for Sydney (New South Wales), London (Greater London), Toronto (Ontario), Berlin (Berlin), Dublin (Leinster), and Paris (Ile-de-France) was consistent with the unadjusted incidence data calculated in Fig. 1A.

A Pearson’s correlation coefficient analysis was conducted to assess the potential effect of mask users per 100 protestors, GSI, and testing per 1000 persons 4 weeks post-protests on the incidence of COVID-19 at 4 weeks post protests (Table 6, Addendum 1). No statistical significance was found between COVID-19 incidence and protest factors.

## Discussion

We showed that in most cities studied, the protests were not associated with increased COVID-19 epidemic activity. There was relatively high mask use at all protests, with over 80% of protestors wearing masks in 8 of the 12 cities analyzed. This is reassuring and suggests that most protesters behaved responsibly by wearing masks and that outdoor mass gatherings with high mask use may not pose a high risk. This is consistent with a study on 315 US cities where BLM protests took place, which found no evidence to suggest that urban protests reignited COVID-19 case growth at two incubation periods post-protest [40].

The resurgence of COVID-19 in Melbourne (Victoria) occurred 4 weeks post the initial protest date and has since been linked by genetic epidemiology to a breach in hotel quarantine of return travelers, rather than to protests [41]. It was reported that at least one of the positive persons identified from the Melbourne (Victoria) protests developed symptoms after the protest but had worn a mask [42, 43], and there is no evidence to suggest anyone became infected at the event. However, due to the inability to trace all the close contacts of the positive protestor, there remains some uncertainty [42, 43].

Contrary to guidance from health authorities to wear masks and avoid use of tear gas, riot control agents such as tear gas have been frequently used during these protests [44]. When case data were adjusted for testing in this study, only Miami (Florida), which involved use of tear gas and had high protest intensity, showed a clear increase after one incubation period post-protest. Tear gas contains 2-chlorobenzalmalononitrile (CS) and phenacyl chloride (CN), which inhibits breathing by attacking the mucous membranes in the eyes and respiratory system and inducing sneezing, coughing, and mucus production [45]. It may also damage mucosal surfaces and make them more susceptible to viral invasion. A study by the United States (U.S.) Army found that recruits who had been exposed to tear gas were more susceptible to respiratory illnesses like the common cold and the flu and that exposure to riot control agents is positively associated with acute respiratory health outcomes [46]. Studies in China and Italy indicate that not only do other irritants such as smoking and air pollution affect SARS-CoV-2 transmission, but that tear gas could potentially result in the development of severe illness [47]. Tear gas can also cause confusion and panic in a crowd, and people may rip off their masks and touch their faces, increasing risk. There has also been evidence of SARS-CoV-2 viral shedding in tears, contributing to another potential mode of transmission when tear gas is used [48]. There have been strong calls for police to cease using these agents due to the risks associated with the COVID-19 pandemic.

Despite the implications for SARS-CoV-2 transmission, it is difficult to conclusively determine whether the unadjusted rise in COVID-19 cases in Miami (Florida), Columbia (South Carolina), and Dallas (Texas) was related to the mass protests that took place. All three cities involved tear gas and had high protest intensity. None of the other cities showed any epidemic surge within two incubation periods of SARS-COV-2. It is important to note that outdoor mass gatherings have a lower risk of transmission than indoor ones, because respiratory aerosols dissipate outdoors, and the risk may be reduced even more by mask wearing [49]. The role of face masks outdoor not only acts as a physical barrier against SARS-CoV-2 but also maintains the temperature of the upper respiratory tract in cold environmental outdoor conditions, thus supporting the innate immune system of upper airways against pathogen threats, and there is increased evidence of the effectiveness of mask use in indoor settings for prevention of SARS-CoV-2 infection [22, 50]. Protests also occurred in summer in Europe and America, and winter in Australia, where winters are mild. The outdoor settings, warmer weather and importance of ventilation (and possibly sunlight) in aerosol transmission, may also have contributed to the lack of an epidemic surge following protests in some of these cities [51]. However, with the recent emergence of the highly transmissible Omicron variant, the risk of outdoor transmission may be higher.

The epidemiology of COVID-19 is influenced by many other factors than protests. In cities such as New York, Sydney (New South Wales), London (Greater London), Toronto (Ontario), Berlin (Berlin), Dublin (Leinster), and Paris (Ile-de-France), a flattening of the curve was seen, despite mass protests taking place. This may be due to other social measures being used at the time. Some cities such as New York (New York) had already passed a large epidemic peak prior to the protests, which may have resulted in more caution being used by the population. A study of the relationship between the George Floyd protests and COVID-19 cases in 8 states in the United States of America showed that positive growth infection rates after the easing of restrictions and the reopening of economies was significant in all states analyzed; however, significant growth after the protests was seen in only 6 of the eight states analyzed [52]. The two states that did not have significant growth rate were recognized as COVID-19 hotspots 2 weeks following the protests and so researchers could not conclusively relate the George Floyd protests to a rise in COVID-19 cases [52]. However, other factors such as the GSI [29], use of tear gas, testing rates, and mask use were not accounted for in that study.

The use of public transport to attend protests may pose a greater risk than the actual protest, because small, enclosed spaces (such as a bus or train carriage) with poor ventilation facilitates transmission [53]. A study of data obtained by cell phone tracking suggests that in cities with large protests, the time spent at home by the general population during the protest period increased, possibly counteracting any surge in COVID-19 cases by protestors themselves [40]. As defined by our inclusion criteria, the protests also occurred at a time when states were allowing the reopening of economies, so other factors may have influenced transmission risk.

A potential limitation of this study is that protests were not limited to one protest per city and continued for weeks after the initial protest date. In addition, events, and behaviors other than protests, and measures other than mask use would have all been at play and influencing the epidemic trajectory in each city, including spatial and geographical factors [54]. We accounted as best we could for this by using the GSI [29]. We also accounted for different ascertainment of infection by examining testing rates, given cities with higher testing may have more complete case ascertainment. We adjusted for this using testing rates to standardize the epidemic curves for each city. It is difficult to calculate precise incidence rates due to high rates of asymptomatic COVID-19 infections. It is also important to note that a potential limitation is that our study was based on open-sourced data, which may be affected by inter-state variations in data publishing policies. Whilst photo-epidemiological methods have previously been used to assess face mask use in mass gatherings where other methods of assessment are difficult and can serve as a rapid and crude assessment tool during crises [30–32], it is also dependent on open-sourced data and images taken at different time points of the protest, capturing a different proportion of protestors, may produce different results. This study did not include police or observers in the persons counted. Protestors may also not be residents in that city and surrounding area; hence, the incidence of COVID-19 in a particular city (and surrounding area) may have been underestimated. A relevant proportion of demonstrators could have been easily travelling far to reach the city and join the protests. We accounted for this by including county data in the analysis. Nonetheless, it is very difficult to evaluate the risk of COVID-19 following protests given the sudden nature of these events and the lack of ability to conduct planned, prospective research. Given the limitations in the study design, we addressed as many confounders as possible by collecting data on other factors which may influence the epidemic trajectory, such as quality of face masks (cloth, surgical N95), consistency of mask use, stringency of government control measures, testing, protest intensity, and use of tear gas; however, SARS-CoV-2 transmission is complex and depends on a variety of different factors. It is also important to note the great variability of protection provided by masks for SARS-CoV-2 transmission [55].

Whilst a rise in epidemic activity post-protest cannot definitely be attributed to the protest, the absence of an epidemic surge within two incubation periods of the protest is indicative that the protests did not have a major influence on epidemic activity. In cities where there was a resurgence within two incubation periods, the effect of protests on this resurgence cannot be ruled out. Furthermore, it has been documented that a more pressing health concern around protests is the injury of protestors and medical staff as a result of excessive crowd control measures [44]. It is important to note that the protests occurred at a time when vaccination was not available, but mask use was high. Social gatherings among unvaccinated public have been associated with increased COVID-19 infections [56]. The findings are not applicable to Omicron, which is far more transmissible, and may have a greater risk of outdoor transmission [57, 58]. A multilayered prevention approach needs to be adopted for any future events of this nature as all mass gatherings will not generate equal risks of SARS-CoV-2 transmission [59]. Risk factors in these settings are dependent on a variety of factors, and public health officials need to apply risk mitigation methods balanced across many different factors including crowd size, duration of the gathering, crowd/urban density, and preventative interventions such as mandated mask use.

## Conclusion

Despite the concerns that outdoor protests may increase SARS-CoV-2 transmission in 2020, we showed that most of the BLM protests analyzed here did not result in a rise in COVID-19 cases in 2020. In Miami, where there was a resurgence within one incubation period, the effect of protests, high protest intensity, and use of tear gas on this resurgence cannot be ruled out. Tear gas should not be used at protests. Preventive measures such as mask use and physical distancing play a role in mitigating transmission of COVID-19. With the globally circulating highly transmissible Alpha, Delta, and Omicron variants, layered interventions such as mandated mask use, physical distancing, testing, and vaccination should be applied for mass gatherings in the future.

### Supplementary Information

Below is the link to the electronic supplementary material.

Supplementary file (XLXS 139 KB)

## Data Availability

Supplied in the supplementary material.

## Addendum 1

Table 4

**Table 4:** Inclusion criteria for cities to be analyzed in this study

Number of protestors/protests	Population Size	Release of restrictions	Use of tear gas
> 50	> 80,000	At least 2 weeks before protest date	Yes/no

Table 5

**Table 5:** Descriptive characteristics of protest images used in the analysis

City	State	Country	Number of images counted	Standard deviation
Lisbon	Estremadura	Portugal	20	3.7
Melbourne	Victoria	Australia	21	6.8
New York	New York	USA	21	8.7
Toronto	Ontario	Canada	20	16.1
Miami	Florida	USA	21	8.3
Columbia	South Carolina	USA	21	12.7
Dallas	Texas	USA	20	14.9
Berlin	Berlin	Germany	20	16.4
London	Greater London	England	20	12.8
Paris	Ile-de-France	France	20	16.3
Sydney	New South Wales	Australia	25	8.9
Dublin	Leinster	Ireland	20	23.4

Table 6

**Table 6:** Correlation coefficient of COVID-19 incidence: a univariate analysis

Variable	Correlation Coefficient		
Testing per 1000 at 4 weeks post-protest	0.0395		
GSI	0.0593		
Mask users per 100	0.0287		

## Addendum 2. Resources

**Table:**

City	Testing sources	Data on cases	Tear gas link
New York City, NY, USA	https://covidtracking.com/data	https://systems.jhu.edu/	https://www.themarshallproject.org/2020/06/03/masks-on-fists-up-scenes-from-new-york-city-s-protests-against-police-violence
Columbia, SC, USA	https://covidtracking.com/data	https://systems.jhu.edu/	https://www.postandcourier.com/news/tear-gas-disperses-protesters-in-columbia-as-city-council-extends-curfew/article_ae851268-a36c-11ea-8ccc-a7b9414ee811.html
Dallas, TX, USA	https://covidtracking.com/data	https://systems.jhu.edu/	https://www.dallasnews.com/news/2020/05/30/protesters-at-dallas-city-hall-say-no-more-and-focus-message-on-police-brutality/
Miami, FL, USA	https://covidtracking.com/data	https://systems.jhu.edu/	https://miami.cbslocal.com/2020/05/30/large-march-held-in-downtown-miami-joining-nationwide-protests/
Toronto, ON, Canada	https://ourworldindata.org/coronavirus-testing	https://www.toronto.ca/home/covid-19/covid-19-latest-city-of-toronto-news/covid-19-status-of-cases-in-toronto/	––-
London, Greater London, UK	https://ourworldindata.org/coronavirus-testing	https://data.london.gov.uk/dataset/coronavirus--covid-19--cases	https://www.youtube.com/watch?reload=9&v=0bNvoz6-FGo
Dublin, Leinster, Ireland	https://ourworldindata.org/coronavirus-testing	https://covid19ireland-geohive.hub.arcgis.com/	––-
Sydney, New South Wales, Australia	https://data.nsw.gov.au/data/dataset/covid-19-cases-by-location	https://data.nsw.gov.au/data/dataset/covid-19-cases-by-location	––-
Melbourne, Victoria, Australia	https://www.dhhs.vic.gov.au/victorian-coronavirus-covid-19-data	https://www.dhhs.vic.gov.au/victorian-coronavirus-covid-19-data	––-
Paris, Ile-de-France, France	https://ourworldindata.org/coronavirus-testing	https://dashboard.covid19.data.gouv.fr/suivi-des-tests?location=FRA	https://www.youtube.com/watch?v=Ps7RbmVMWzY
Berlin, Berlin, Germany	https://ourworldindata.org/coronavirus-testing	https://www.rki.de/EN/Home/homepage_node.html	––-
Lisbon, Estremadura, Portugal	https://ourworldindata.org/coronavirus-testing	https://covid19.min-saude.pt/relatorio-de-situacao/	––-
